# Proactive Inhibition Activation Depends on Motor Preparation: A Single Pulse TMS Study

**DOI:** 10.3389/fpsyg.2018.01891

**Published:** 2018-10-05

**Authors:** Stefania C. Ficarella, Lorella Battelli

**Affiliations:** ^1^Center for Mind/Brain Sciences, University of Trento, Rovereto, Italy; ^2^Center for Neuroscience and Cognitive Systems@UniTn, Istituto Italiano di Tecnologia, Rovereto, Italy; ^3^Laboratoire de Neurosciences Cognitives, Aix-Marseille Univ, CNRS, LNC, UMR 7291, Marseille, France; ^4^Department of Neurology, Beth Israel Deaconess Medical Center, Berenson-Allen Center for Noninvasive Brain Stimulation, Harvard Medical School, Boston, MA, United States

**Keywords:** action inhibition, proactive, TMS, cognitive control, MEP, cortico-spinal excitability, motor preparation

## Abstract

In everyday life, environmental cues are used to predict and respond faster to upcoming events. Similarly, in cueing paradigms (where, on cued trials, a cued target requires a speeded response), cues are known to speed up response times (RTs), suggesting that motor preparation has occurred. However, some studies using short cue-target intervals (<300 ms) have found slower RTs on cued, compared to uncued trials (namely, the “paradoxical warning cost”). One explanation of this paradoxical effect is proactive inhibition, a motor gating mechanism that prevents false alarms, also called “the default state of executive control.” Alternative hypotheses claim that, with such short cue-target delays, participants cannot fully prepare the motor response, thus producing slower RTs. In studies of action inhibition, it is often assumed that participants prepare a response on each trial, a prerequisite to induce and measure (proactive) motor inhibition. In this study, we psychophysically manipulated stimulus’ duration in a simple RT task, and measured a duration threshold at which participants responded on time on 80% of the trials. When participants are tested at their stimulus’ duration threshold, they are more likely to prepare the motor response on each trial. Furthermore, we directly measured participants’ readiness to respond by recording transcranial-magnetic stimulation (TMS)-elicited motor evoked potentials (MEPs), a direct measure of corticospinal excitability. Participants performed cued and uncued trials on a simple RT task with short cue-target intervals. We expected lower MEPs’ amplitude on cued than uncued trials with short cue-target intervals, as it would be predicted by the proactive inhibition account. However, when conditions are equated so that motor preparation is induced both under cued and uncued trials, the paradoxical warning cost disappears, as RTs were always faster on cued than uncued trials. Moreover, MEPs recorded from the task-relevant muscle were never suppressed at target onset compared to baseline, a result that does not support the proactive inhibition hypothesis. These results suggest that proactive inhibition is not active by default and that its activation depends on motor preparation.

## Introduction

In everyday life, we often must quickly react to stimuli in the environment, such as catching a ball unexpectedly thrown at us. To efficiently and promptly react to unexpected events, we seek for signals in the environment that might help us predict upcoming stimuli. Basketball players look for changes in the opponent’s gaze to predict the direction of his/her next move. To investigate the effects of cues on motor preparation and inhibition, instructed delayed simple or choice RT tasks are used in the lab. In simple RT tasks, a non-informative, alerting cue [warning signal (WS)] informs participants *when* a planned action should be executed (“time preparation”), while in choice RT tasks, an informative cue indicates which response to perform at target onset, leading to what is called “event preparation” (Requin et al., 1991; Hasbroucq et al., 1999). The cues or WSs used in these paradigms are known to speed up RTs (Woodworth and Schlosberg, 1954; Näätänen, 1970; Niemi and Näätänen, 1981), an effect known as “alerting benefit.” Possible mechanisms responsible for this effect include sensory/perceptual processes, action selection, and/or motor processes (Fecteau and Munoz, 2007). Whatever the underlying cause of the alerting benefit effect, executive control functions are thought to be necessary to prevent the premature execution of the, supposedly prepared, responses (Greenhouse et al., 2015; Duque et al., 2017). Specifically, while a “competition-resolution” process is thought to contribute to action selection in choice RT tasks by inhibiting alternative task-irrelevant responses (Burle et al., 2004; Ridderinkhof et al., 2004), proactive inhibition would exert “impulse control” to prevent false alarms (Duque et al., 2010). Reactive forms of inhibitory control are thought to be engaged at the presentation of stop signals or NoGo stimuli (in stop signal and Go/NoGo paradigms, respectively; Aron, 2011; Swick et al., 2011), whereas proactive inhibitory mechanisms are activated “in anticipation of stimulation when the situation is unpredictable” (Criaud et al., 2017).

To study proactive inhibition, previous studies have adopted cueing (Wardak et al., 2012; Smittenaar et al., 2013) or Go/NoGo paradigms (Criaud et al., 2012; Albares et al., 2014) including blocks of randomly mixed cued/uncued trials or Go/NoGo trials, in which the stimulus identity (cue vs. target and Go vs. NoGo, respectively) cannot be predicted at trial onset. Under such conditions of uncertainty, the proactive inhibition account predicts that motor inhibitory mechanisms will prevent the automatic execution of responses to the first appearing event (Boulinguez et al., 2008; Jaffard et al., 2008), slowing down responses and suppressing motor evoked potentials MEPs, an index of corticospinal excitability (CSE) that indicates readiness to respond (Claffey et al., 2010; Cai et al., 2011). It is important to note that, in order to distinguish action inhibition from an absent motor preparation, the task should be designed to motivate participants to prepare the response(s) on each trial (e.g., by manipulating the Go stimulus probability on Go/NoGo tasks, see Wessel, 2017 and Ficarella and Battelli, in this issue for a review) and/or direct measures of motor preparation should be recorded (e.g., MEPs).

Importantly, most of the studies on action inhibition lack a crucial control condition of a pure block of uncued trials (or a block including only Go trials, for the Go/NoGo task), in which participants know that only the target will appear and proactive inhibition is not required (Jaffard et al., 2007). Finally, few studies have investigated warning effects using cue-target intervals shorter than 500 ms, and they found slower RTs for short compared to long intervals (Nickerson, 1965; Sanders, 1972; Requin et al., 1991; Lebon et al., 2015). Some studies have found a “paradoxical warning cost” when short cue-target intervals (shorter than 300 ms) are used in a simple RT task, with cued trials eliciting longer RTs than the control block of uncued trials (Jaffard et al., 2007; Boulinguez et al., 2009). This effect has been interpreted as due to a delay (of about 300 ms) in proactive inhibition deactivation, triggered by stimulus identification. This proactive inhibition interpretation of behavioral effects, derived from EEG (Boulinguez et al., 2009) and fMRI (Jaffard et al., 2008) data, should be corroborated by more direct measures of ongoing, and inhibited, motor preparation. In fact, while neuroimaging studies have found activations (and EEG components) consistent with the involvement of proactive inhibition circuits (including medial prefrontal and inferior parietal cortex), they still do not provide unequivocal evidence of motor suppression.

In this study, we directly test the hypothesis whether proactive inhibition causes the RT cost on cued trials, by measuring TMS-elicited MEPs, during a simple RT task. Since we aimed at targeting the proactive action inhibition mechanisms, we chose to use a simple, instead of choice, RT task to exclude potential confounds associated with action selection-related inhibitory mechanisms (Duque et al., 2010). We devised a new task in which the duration of the RS resulted from an individually calibrated speed threshold, measured in a preliminary psychophysical task. Crucially, the (short) RS duration, together with two fast SOAs, likely ensured that participants prepared the response at the beginning of each trial. If proactive inhibition is activated to prevent false alarms and “is maximum when the target appears soon after the WS” (Jaffard et al., 2008), we should expect MEPs of the task-relevant muscle to be suppressed at RS onset, compared to baseline (trial onset). If proactive inhibition is activated when there is uncertainty regarding stimulus identity (mixed block condition) and when a cue precedes the RS (pure block of cued trials), we should expect to find MEP suppression at RS onset. Moreover, if proactive inhibition deactivation takes around 300 ms to be implemented (the paradoxical warning cost), we should predict suppression of the MEPs for the shortest SOAs only. Conversely, a lack of MEPs suppression at RS onset would not support the proactive inhibition account, suggesting that proactive inhibition is not active by default, and that its activation is context dependent.

## Materials and Methods

### Participants

Twenty-seven university students (14 females; mean age 22.5 ± 3.8 years) voluntarily participated in the study. All participants were right-handed, according to the modified Edinburgh Handedness Inventory (Oldfield, 1971) and they were screened for TMS inclusion/exclusion criteria. They had normal or corrected-to-normal vision. The study was approved by the ethical committee of the University of Trento. All participants gave their written informed consent and were paid for their participation.

### Experimental Procedure

First, the rMT of each participant was assessed using TMS. TMS (biphasic) pulses were delivered using a 70 mm figure-8-coil connected to a Magstim Rapid^2^ (Magstim Co., United Kingdom) and MEPs were registered using the software LabChart 7 (ADInstruments, Oxford, United Kingdom) at a sampling frequency of 10 kHz, applying a bandpass filter between 20 and 2500 Hz. Participants’ MEPs were recorded from the FDI and the ADM of the right, dominant hand, using Ag/AgCl surface electrodes (6 mm diameter), with the ground electrode placed on the participants’ wrist. The left hand area (M1) was found by eliciting visible twitches on the contralateral hand muscles and the hot spot for the FDI was assessed by measuring the rMT, indexed as the lowest intensity of stimulation able to elicit MEPs of >50 μV peak-to-peak amplitude on five out of 10 consecutive pulses (Rossini et al., 1994). TMS stimulation intensity was then set to 110% of the rMT (mean stimulation output: 66 ± 11% of the maximal stimulator’s intensity). Participants wore an elastic cap on which the position of the coil was drawn. Each participant was then randomly assigned to one of two groups in which 20 MEPs at rest were recorded either at the beginning or at the end of the experiment. Participants then performed a preliminary *speed threshold task*, during which no TMS was applied. This baseline task was used to psychophysically determine, for each participant, the duration threshold (see next paragraph) of the target subsequently used in the simple RT task. In the simple RT task, the hotspot for the task-relevant FDI muscle was stimulated with TMS and MEPs were recorded from both muscles (FDI and ADM). Participants responded by abducting the right index finger to press the space bar of the keyboard placed vertically next to their arm, with the keys facing the right hand. They were asked to keep the muscles relaxed before and after each response. Throughout each session (about 2 h), subjects were seated on a comfortable chair at a distance of 57 cm from the computer screen, with their chin on a chinrest. The task was presented on a 22″ Samsung 2233RZ LCD monitor running at 120 Hz on a Windows 7 machine running Matlab 7.2 and Psychtoolbox 2.0 experimentation presentation software.

### Speed Threshold Task

**FIGURE 1:**
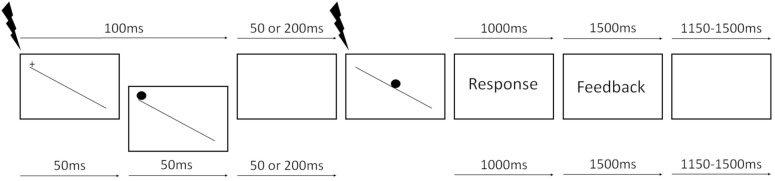
Trial structure (for showing purposes colors are inverted, the background is black and the plane and marble are white). Top and bottom arrows represent uncued and cued trials time intervals, respectively. Black lightning bolts represent TMS pulses, delivered either at the presentation of the fixation cross (baseline) or at RS onset (see section “Materials and Methods” for details).

Each trial started with a white fixation cross displayed at the top of a tilted plane (e.g., leftmost panel on **Figure 1**) for 100 ms. The plane consisted of a white line, 0.35 thick and 27.8 long degrees of visual angle, with a luminance of 204.5 cd/m^2^, tilted at an angle of 30°, running from the upper left to the bottom right quadrant of the screen. Subsequently, a white static marble (cue), 3.55° in diameter, was presented for either 50 or 200 ms randomly across trials. The marble then started rolling down the tilted plane at a fixed velocity. The rolling marble was the RS, hence requiring participants to respond. The total duration of the SOA from trial onset was therefore either 150 or 300 ms. The marble was always presented at the top of the plane, at the same location as the fixation cross, hence on the top-left side of the monitor. All stimuli were presented on a black background (0.2 cd/m^2^). Participants were asked to wait for the marble to start moving (RS) and then abduct their right index finger to press the space bar as quickly as possible to stop the marble. We used a 3-up-1-down staircase procedure to determine, for each participant, the threshold speed at which they were able to correctly stop the marble before falling off the plane on 80% of the trials. Participants performed five training trials, and the staircase stopped once six reversals or 100 trials were completed and the speed threshold was estimated. Average marble speed was 0.092 ± 0.011° of visual angle/second and the average duration of the marble presentation, once it started moving (after the variable SOA), was 293 ± 36 ms. The marble’s speed threshold measured individually was then used in the simple RT task.

### TMS Experiment

In the simple RT task, participants performed three blocks: two pure blocks of *cued* and *uncued* trials and a mixed block, in which these two trial types were randomly intermixed. The pure block of uncued trials condition served as a control based on the prediction that the activation of proactive inhibition mechanisms was not required. The order of presentation of the three blocks was pseudorandomized so that each block sequence was equally represented in the group of participants. Each trial started with a white fixation cross at the top of the tilted plane for either 50 ms on cued trials or 100 ms on uncued trials (see **Figure 1**). On cued trials, a white marble replaced the fixation cross for 50 ms, representing a non-informative WS, namely, the “cue.” After a randomly chosen inter-stimulus-interval of either 50 or 200 ms, during which a black screen was presented, the target white marble appeared on top of the tilted plane and immediately started rolling down at the threshold speed measured during the *speed threshold* task. Each trial duration was the same for cued and uncued trials, and the time between the appearance of the fixation cross and target presentation was either 150 (short SOA) or 300 ms (long SOA) for both conditions. Participants were asked to always wait for the RS, keeping the muscles relaxed, and then abduct the index finger as quickly as possible to press the spacebar and stop the marble. Once the marble reached the end of the plane, a black background was displayed for 1000 ms during which late responses were recorded. At the end of each trial, a feedback message was displayed for 1500 ms with the text “Too early” if they responded before the target, “Stopped” if they blocked the marble before it fell off the plane, or “Too late” if they pressed the spacebar after the marble fell off the plane. The ITI was randomly chosen between 1150 and 1500 ms, during which a black background was presented. The total duration of each trial was variable, depending on the SOA, the speed of the ball, the RTs, and the ITI, between 4400 and 5000 ms (see **Figure 1**). On pure blocks (no uncertainty condition), participants performed eight training trials and 256 valid trials with a break every 64 trials, in which subjects rested their eyes while keeping the head on the chinrest. On the mixed block (uncertainty condition), participants performed 16 training trials and 512 valid trials, with short breaks every 64 trials and a longer break halfway through the block. Single pulse TMS was used to stimulate the hotspot corresponding to the right FDI muscle, while MEPs were recorded from FDI (task-relevant) and ADM (task-irrelevant) muscles. TMS pulses were delivered once every two trials, to have enough time between two consecutive pulses (at least 8 s) and prevent cumulative effects of the pulses. On TMS trials, the timing of the stimulation was pseudorandomly chosen between two possible time points: at the presentation of the fixation cross (baseline) or at RS onset.

### Data Analysis

We compared, within subjects, RTs, MEPs, and number of errors (false alarms, anticipations, and late responses) across four task conditions: cued pure, uncued pure, cued mixed, and uncued mixed. Within each condition, trials were divided according to the SOA (short vs. long) and, for the MEP analysis, according to TMS pulse time (baseline vs. RS onset). Since a TMS pulse was delivered every two trials, we included 32 trials per SOA^∗^TMS pulse condition and, for RTs, 64 trials per SOA within each task condition. Anticipations (RTs < 100 ms), false alarms (responses during the SOA), and late responses (after the marble reached the end of the plane) were considered errors, and they were analyzed using a non-parametric ANOVA (Friedman test) for dependent samples with Wilcoxon *post hoc* test, since the criteria for parametric tests were not met. Since RTs were modulated by the presence of a TMS pulse, as demonstrated by three one-way repeated measures ANOVAs, one for each task block, to compare average RTs across TMS conditions (no pulse, baseline pulse, and target onset pulse), only no-TMS pulse trials were used to calculate the average RTs. Average RTs from the eight cue-by-task conditions were analyzed with a three-way repeated measures ANOVA with Fisher’s LSD *post hoc* test, including factors: cue (no cue vs. cue), SOA (150 vs. 300 ms), and block (pure vs. mixed). Given the task’s difficulty, late responses were relatively frequent and they were included in the analysis of RTs and MEPs; however, the results do not change including on-time responses only. Trials in which noise (amplitude > 50 μV) was present in the 100 ms period before the TMS pulse were discarded from the MEP analysis (16 ± 11, 19 ± 11, and 19 ± 12% of the total trial number for the uncued pure, cued pure, and mixed block, respectively). MEPs amplitudes for the eight cue-by-task conditions recorded at RS onset were log-transformed. One way-repeated measures ANOVAs with Fisher *post hoc* test were used to compare baseline MEPs across conditions and baseline vs. resting MEPs. Since baseline MEP amplitudes were similar across conditions, the average baseline value was used for subsequent analyses. Student’s *t*-tests were used to compare baseline MEPs vs. RS onset MEPs. Finally, to take into account the variability of baseline MEPs, we normalized them by dividing, for each participant, the raw mean MEP amplitude at RS onset by baseline values, and then log-transformed them. Normalized MEPs from the eight cue-by-task conditions were analyzed with three-way repeated measures ANOVAs with Fisher’s LSD *post hoc* tests, including factors: cue (no cue vs. cue), SOA (150 vs. 300 ms), and block (pure vs. mixed). Whenever required, the Greenhouse–Geisser correction was applied and results will be reported with the uncorrected degrees of freedom along with the Greenhouse–Geisser epsilon (Picton et al., 2000). For all analyses, the significant level was set to α = 0.05. A summary of all the analyses we carried out, divided by type of data, is presented in **Table 1**.

**Table 1:** Summary of the statistical analyses.

Data	Statistical test	*Post hoc* test
Errors	Friedman	Wilcoxon
Average RTs	Three-way repeated measures ANOVA	Fisher’s LSD
Baseline vs. rest MEPs	One-way repeated measures ANOVA	Fisher’s LSD
Baseline MEPs across conditions	One-way repeated measures ANOVA	n.a.
Target MEPs vs. baseline	Student’s *t*-test	n.a.
Normalized target MEPs across conditions	Three-way repeated measures ANOVA	Fisher’s LSD
Average RTs without and with TMS	One-way repeated measures ANOVA	n.a.


*Note that only main significant results are reported in the results section. n.a., not applicable.*

## Results

### Errors

**FIGURE 2:**
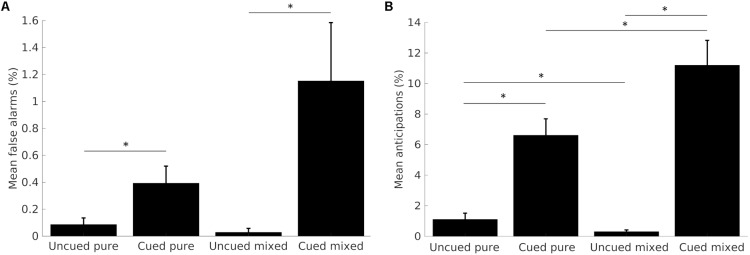
Mean percentages of false alarms **(A)** and anticipations **(B)** across task conditions. Data from the two SOA conditions are merged together. Error bars represent the standard error from the mean. Asterisks show significant differences (*p* < 0.05).

Early responses made during the SOA (false alarms), or in less than 100 ms (anticipations) were considered, together with late button presses delivered after the marble fell off the plane, as errors, and analyzed with non-parametrical ANOVAs with Wilcoxon *post hoc* corrections. Although we separated false alarms from anticipations for clarity, they likely result from the same underlying mechanisms, as indeed demonstrated by the similar trend across conditions (**Figure 2**).

#### False Alarms

Participants committed very few false alarms, and almost none in short SOA trials. The comparison of the total number of false alarms across the four task conditions yielded significant results (*p* < 0.001, *df* = 3). Participants committed significantly more false alarms on cued, compared to uncued trials, both in the pure (*Z* = -2.230, *p* < 0.05) and the mixed block condition (*Z* = -3.237, *p* < 0.005). Differences across blocks were not significant. Results are shown in **Figure 2A**.

#### Anticipations

Participants committed almost zero anticipations in short SOA trials; therefore, we analyzed the total number of errors. The comparison of anticipations’ frequency across the four task conditions was statistically significant (*p* < 0.001, *df* = 3). The *post hoc* analysis showed that participants committed significantly more anticipations on cued, compared to uncued trials, for both the pure (*Z* = -4.380, *p* < 0.001) and the mixed block (*Z* = -4.460, *p* < 0.001). Moreover, when comparing blocks, results show a significantly greater number of errors in the cued mixed, compared to the cued pure trials (*Z* = -2.676, *p* < 0.05), whereas uncued mixed trials elicited less anticipations than the uncued pure trials (*Z* = -2.469, *p* < 0.05). Results are shown in **Figure 2B**.

#### Late Responses

**FIGURE 3:**
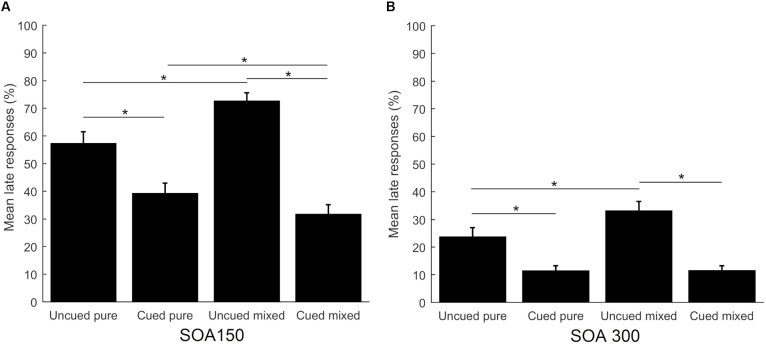
Mean percentage of late responses across task conditions for the short SOA **(A)** and long SOA **(B)**. Error bars represent the standard error. Asterisks show significant differences (*p* < 0.05).

Separate analyses were conducted to compare the percentage of late responses across the four task conditions, separately for the two SOAs. They both yielded significant results (*p* < 0.001, *df* = 3 for both SOAs). *Post hoc* comparisons were significant for both SOA conditions: uncued pure vs. cued pure (*Z* = -3.785 for short SOA and *Z* = -4.363 for long SOA, *p* < 0.001), uncued mixed vs. cued mixed (*Z* = -4.520 for short SOA and *Z* = -4.543 for long SOA, *p* < 0.001), uncued pure vs. uncued mixed (*Z* = -3.622, *p* < 0.001 for short SOA and *Z* = -3.203, *p* = 0.001 for long SOA), while the comparison cued pure vs. cued mixed was only significant for the short SOA condition (*Z* = -2.619, *p* < 0.05). Additionally, the short SOA condition elicited significantly more late responses in all task conditions (*p* < 0.001). Results are shown in **Figure 3**.

### Response Times

**FIGURE 4:**
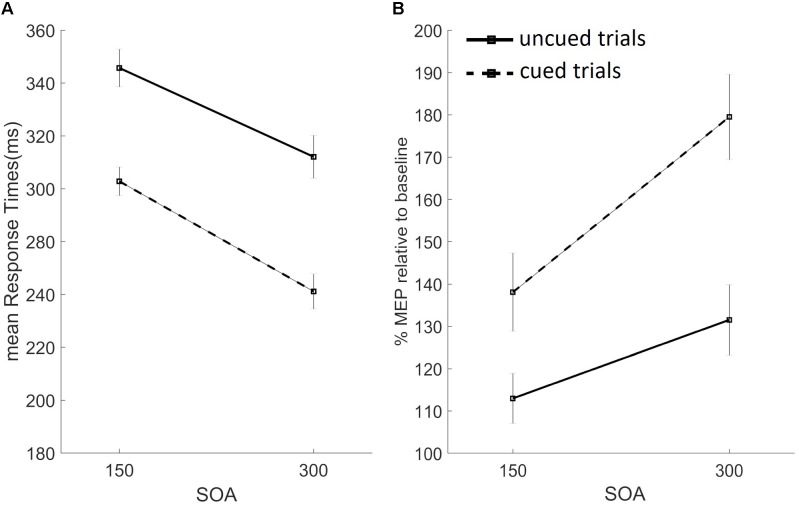
Results from the uncued (black filled lines) and cued (black dashed lines) trials, for the short (150 ms) and long (300 ms) SOA conditions are presented. Data from the pure and mixed blocks were averaged. **(A)** Mean response times in milliseconds. **(B)** Percentage of mean MEP amplitudes at target onset, relative to baseline, for the FDI muscle. Error bars represent the standard error.

We analyzed RTs using a three-way repeated measures ANOVA with factors: cue (no cue vs. cue), SOA (150 vs. 300 ms), and block (pure vs. mixed). Significant main effects were found for factors cue [*F*(1,26) = 234.157, *p* < 0.01] and SOA [*F*(1,26) = 213.687, *p* < 0.01], but not for block [*F*(1,26) = 3.105, *p* = 0.09]. As depicted in **Figure 4A**, RTs were slower on uncued than cued trials, and on trials with the short vs. long SOA. Significant two-way interactions were found for cue-by-SOA [*F*(1,26) = 7.636, *p* < 0.05], cue-by-block [*F*(1,26) = 20.980, *p* < 0.001], and SOA-by-block [*F*(1,26) = 10.619, *p* < 0.05], while the three-way interaction did not reach significance. Trials with the long vs. short SOA sped up RTs. This SOA effect interacted with both cue type, inducing a stronger alerting benefit (faster RTs on cued vs. uncued trials) on trials with the long SOA (cue-by-SOA interaction), and with block type (stronger SOA effect of pure blocks), as per the SOA-by-block interaction. Finally, RTs were slower in the mixed vs. pure block, only on uncued trials, as confirmed by the significant cue-by-block interaction (baseline shift effect).

Average RTs on TMS trials were generally faster, as confirmed by three one-way repeated measures ANOVAs, one for each task block, used to compare average RTs across TMS conditions (no pulse, baseline pulse, and target onset pulse). Significant differences were found for all three blocks: pure uncued [*F*(2,52) = 61,410, *p* < 0.001], pure cued [*F*(2,52) = 52.846, *p* < 0.001, ε = 0.793], and mixed [*F*(2,52) = 51.112, *p* < 0.001, ε = 0.680] block. A visual inspection of these data revealed a similar pattern as the average RTs from no-TMS trials depicted in **Figure 4A**, in which data from the two blocks were merged as the factor block was not significant.

### Motor Evoked Potentials

Baseline MEPs across conditions did not differ, as confirmed by a one-way repeated measures ANOVA neither for the FDI [*F*(3,78) = 1.149, *p* = 0.33, ε = 0.042] nor for the ADM [*F*(3,78) = 0.433, *p* = 0.65, ε = 0.016] muscles; therefore, they were averaged for further analyses. We used separate two-tailed Student’s *t*-tests for each of the eight task conditions to compare MEPs recorded at target onset with the (averaged) baseline value. FDI MEP amplitudes at target onset were significantly higher than baseline for cued mixed conditions both at the short [*t*(26) = 3.311, *p* = 0.003] and long [*t*(26) = 3.821, *p* = 0.001] SOAs, and cued pure at the long SOA [*t*(26) = 3.810, *p* = 0.001]. The same analysis on the task irrelevant ADM muscle did not show any significant difference.

The proactive inhibition account predicts that, on trials of the mixed block and the pure cued block, the task-relevant muscle should be maximally suppressed at RS onset. In our study, average FDI MEPs were higher than at baseline, hence not supporting the proactive inhibition hypothesis.

To investigate the effects of task conditions while taking into account the variability of baseline FDI MEPs, we normalized target MEPs by dividing, for each participant, the mean MEP amplitude at RS onset by baseline values, and then log-transformed them, separately for the two muscles. Two three-way repeated measures ANOVAs with factors cue (no cue vs. cue), SOA (150 vs. 300 ms), and block (pure vs. mixed) were used. No main effects were found for the task-irrelevant muscle ADM [cue: *F*(1,26) = 0.173, *p* = 0.68; SOA: *F*(1,26) = 0.242, *p* = 0.63; block: *F*(1,26) = 0.256, *p* = 0.62]. Two-way interactions and the three-way interaction were also not significant. Conversely, significant effects were found for the task-relevant muscle FDI for factor cue [*F*(1,26) = 12.534, *p* < 0.05] and SOA [*F*(1,26) = 8.541 m *p* < 0.05], but not for the factor block [*F*(1,26) = 0.458, *p* = 0.5]. As depicted in **Figure 4B**, FDI MEPs were higher on cued vs. uncued trials, and for trials with the long vs. short SOA. The only significant two-way interaction we found was SOA-by-block [*F*(1,26) = 4.890, *p* < 0.05], and the three-way interaction did not reach statistical significance. The significant two-way interaction suggests that the SOA effect (higher MEP amplitude on trials with the long SOA) was modulated by block type, being the effect stronger on pure blocks. The percent change of normalized MEPs recorded at target onset, relative to baseline, for the relevant FDI muscle (averaged from the two blocks) are shown in **Figure 4B**.

Finally, we compared MEPs recorded at rest with MEPs at baseline (trial onset) for the four task conditions and the two muscles. Two repeated measures ANOVAs, one for each muscle, were conducted including the four task conditions and rest MEPs. Results showed no significant differences for the relevant muscle FDI [*F*(4,104) = 0.680, *p* > 0.5, ε = 0.744] and significant differences for the control muscle ADM [*F*(4,104) = 2.843, *p* < 0.5, ε = 0.734]. Pairwise comparisons showed that all ADM MEPs recorded at baseline were significantly smaller than rest MEPs in all conditions (*p* < 0.05, the uncued pure condition showed a trend toward significance, *p* = 0.081).

## Discussion

In this study, we show that, when carefully controlling for inter-individual differences in motor preparation under time-pressure conditions, proactive inhibition is not activated. While warning effects were evident on both behavioral (RTs and error rates) and electrophysiological measures (MEPs), the expected proactive inhibition-induced MEP suppression was not found.

Overall, participants committed very few errors, suggesting that they were able to perform the task as suggested by the speed/accuracy trade-off. We found a warning effect, in that subjects always responded faster on cued trials (main cue effect), they committed more errors (too fast responses) and less late responses. Interestingly, while previous studies found this “alerting benefit” on mixed blocks of cued/uncued trials, they, instead, reported a paradoxical warning cost on pure blocks, with slower RTs on pure cued vs. uncued trials at short SOAs (Jaffard et al., 2007; Boulinguez et al., 2009). In our study, RTs on cued trials were always faster than uncued trials (“alerting benefit,” **Figure 4A**) and no paradoxical warning cost was found. Our crucial psychophysical manipulation of stimulus duration *forced* participants to prepare the response at each trial’s onset, to execute it within the short allotted time. Giving the difficulty of the task, while participants were not able to predict RS onset on uncued trials (as suggested by slow RTs and baseline-levels MEPs recorded at target onset), they likely used cue onset to predict target onset, therefore producing the alerting benefit.

When participants had more time (long SOA condition) to prepare the response, they were faster and MEPs amplitude was higher (significant main SOA effect for both RTs and FDI MEPs). Moreover, the cue-by-SOA interaction found in RTs indicates that the alerting benefit was stronger for trials with the long SOA, in which participants had more time to prepare the response before target onset. Finally, the SOA effect (faster RTs and higher MEPs’ amplitudes on long SOA trials) was stronger on pure blocks, as confirmed by significant SOA-by-block interactions. Participants may have used a more cautious strategy in the mixed block, in which there was uncertainty regarding the upcoming stimulus’ identity, thus explaining the weaker effects of cue and SOA on RTs and MEP amplitudes.

Together, these results argue against the hypothesis that supports proactive inhibition as a default mechanism (Criaud et al., 2012) and suggest that, when motor preparation is induced on each trial, the paradoxical warning cost disappears. We also replicated the “baseline shift” of RTs with slower uncued trials (as well as less anticipations and more late responses) in the mixed block condition, compared to the pure block, confirmed by the significant cue-by-block interaction. Boulinguez et al. (2009) proposed that this effect is due to proactive inhibitory mechanisms, aimed at preventing false alarms, active in the mixed block design only. In line with this hypothesis, we should have expected to find suppressed MEPs on mixed uncued trials. However, we did not find evidence of motor inhibition, since all FDI target MEPs were higher than baseline levels. These results suggest that alternative non-motor factors induced the baseline shift effect (e.g., higher readiness and lower attentional load in pure blocks, for a review see Los, 1996). In the simple RT task adopted in this study, stimuli were always presented at fixation and responses were delivered with the dominant hand, to avoid lateralization effects. Nonetheless, an attention induced modulation on motor preparation could explain the present results, the so-called *motor attention theory* (Rushworth et al., 1997, 2003). In line with this view, we speculate that our participants covertly allocated their attention to the muscles involved in the task on each trial, and that cue onset boosted this attention-induced motor preparation, shortening RTs and enhancing CSE levels.

In our study, the task-irrelevant control muscle ADM was never used throughout the whole experiment and, while we did not find MEPs smaller than baseline in any task condition, baseline ADM MEPs were significantly smaller than at rest. This result is consistent with the inhibition for “competition-resolution” hypothesis (Burle et al., 2004; Ridderinkhof et al., 2004), suggesting that participants might have inhibited ADM activity at the beginning of the experiment, inhibition that was kept constant and not influenced by task conditions.

A potential limitation of this study is that a fixation cross appeared at trial onset instead of being displayed already during the ITI. This manipulation allowed us to compared cued and uncued trials at different SOAs; however, while one might argue it could have acted as cue itself, our RTs for the uncued pure condition were counterintuitively quite slow, even slower than previous studies (Boulinguez et al., 2009), indicating that the fixation cross was not used to predict stimulus (or cue) onset. Additionally, we still found a significant effect of the cue when we averaged RTs, suggesting that the very brief presentation of the fixation cross did not disrupt the task design and likely elicited motor preparation.

## Conclusion

Our data show that proactive inhibition mechanisms are not always active by default and that the execution of a planned action under speed-stressing conditions does not require anticipatory inhibition. Finally, our results stress the importance of monitoring motor preparation levels in action inhibition studies, in order to disentangle the effects of motor inhibition from fluctuations of motor preparation levels.

## Ethics Statement

This study was carried out in accordance with the recommendations of the ethical committee of the University of Trento. The protocol was approved by the ethical committee of the University of Trento. All subjects gave written informed consent in accordance with the Declaration of Helsinki.

## Author Contributions

SF and LB planned the study. SF conducted the study and wrote the manuscript. LB revised the manuscript.

## Conflict of Interest Statement

The authors declare that the research was conducted in the absence of any commercial or financial relationships that could be construed as a potential conflict of interest.

## Abbreviations

ADM: abductor digiti minimi
CSE: corticospinal excitability
FDI: first dorsal interosseous
ITI: inter-trial interval
MEPs: motor evoked potentials
RS: response signal
RTs: response times
rMT: resting motor threshold
SOA: stimulus-onset asynchrony
TMS: transcranial-magnetic stimulation
